# Influence of Plant Growth-Promoting Rhizobacteria (PGPR) Inoculation on Phenolic Content and Key Biosynthesis-Related Processes in *Ocimum basilicum* Under *Spodoptera frugiperda* Herbivory

**DOI:** 10.3390/plants14060857

**Published:** 2025-03-10

**Authors:** Jimena Sofía Palermo, Tamara Belén Palermo, Lorena del Rosario Cappellari, Gerd Ulrich Balcke, Alain Tissier, Walter Giordano, Erika Banchio

**Affiliations:** 1 INBIAS Instituto de Biotecnología Ambiental y Salud (CONICET—Universidad Nacional de Río Cuarto), Campus Universitario, Río Cuarto 5800, Argentina; jpalermo@exa.unrc.edu.ar (J.S.P.); tpalermo@exa.unrc.edu.ar (T.B.P.); lcappellari@exa.unrc.edu.ar (L.d.R.C.); wgiordano@exa.unrc.edu.ar (W.G.); 2 Department of Cell and Metabolic Biology, Leibniz Institute of Plant Biochemistry, Weinberg 3, 06120 Halle (Saale), Germany; gerd.balcke@ipb-halle.de (G.U.B.); alain.tissier@ipb-halle.de (A.T.)

**Keywords:** herbivory, *Ocimum basilicum*, phenylalanine ammonia-lyase, plant growth-promoting rhizobacteria, phytohormone, rhizobacteria, secondary metabolites, *Spodoptera frugiperda*, total phenolic compounds

## Abstract

Plants are naturally subjected to various types of biotic stresses, including pathogenic microorganisms and herbivory by insects, which trigger different signaling pathways and related defense mechanisms. Inoculation with microorganisms, such as plant growth-promoting rhizobacteria (PGPR), can be seen as a form of stress because it triggers a systemic resistance response in plants similar to that caused by insect herbivory. However, these interactions have typically been studied independently, which has limited the understanding of their combined effects. This study examines the effects of *Bacillus amyloliquefaciens* GB03 inoculation and *Spodoptera frugiperda* herbivory on the total phenolic contents of *Ocimum basilicum*. We also analyze the levels of endogenous phytohormones and the activity of phenylalanine ammonia-lyase (PAL), a crucial enzyme involved in the biosynthesis of phenolic defense-related metabolites. The results indicate that the total phenolic content significantly increased only in plants that were both inoculated by GB03 and damaged by larvae. Additionally, PAL activity showed an increase in plants that were damaged by larvae and in those subjected to the combined treatment of larval damage and inoculation with GB03. Regarding phytohormones, in plants damaged by insects, the levels of salicylic acid (SA) increased, regardless of whether they were inoculated or not, while the levels of jasmonic acid–isoleucine (JA-ile) rose in all treatments compared to the control. This study highlights the intricate relationships among beneficial microbes, herbivores, and plant defense mechanisms, emphasizing their potential impact on improving plant resilience and the production of secondary metabolites. Furthermore, understanding the independent effects of PGPR inoculation, beyond its interaction with herbivory, could provide valuable insights into its role as a sustainable alternative for enhancing plant defense responses and promoting crop productivity.

## 1. Introduction

All living organisms, including plants, animals, and microorganisms, experience different forms of biotic stressors throughout their life cycle and must adapt to avoid the effects of these challenging environmental conditions [1]. Extensive research has shown that when plants perceive biotic and abiotic stress, systemic signaling and adaptive responses lead to the accumulation of specialized metabolites (SMs) [2,3,4]. However, even though their biosynthesis requires a significant amount of energy, these specialized compounds offer plants an efficient means of defense against different stressors. The production and storage of SMs influence both plant defense and growth processes, with an impact that depends on the stage of development, type of tissue, and particular stress factors [5].

Among these specialized metabolites, phenolic compounds play a pivotal role in plant defense and adaptation by contributing to structural reinforcement, antimicrobial activity, and oxidative stress responses [6,7]. Under abiotic stress, phenolic compounds mitigate damage caused by extreme light and temperature fluctuations, UV radiation, nutrient imbalances, drought, and flooding. They act as antioxidants, scavenging reactive oxygen species (ROS) to prevent cellular damage, and contribute to the fortification of cell walls, reducing water loss and enhancing mechanical resistance against environmental stressors [8]. In response to biotic stress, phenolic compounds provide multiple layers of defense against pathogens and herbivores [9]. Their antimicrobial properties stem from their ability to disrupt pathogen integrity by altering membrane permeability, inhibiting enzyme activity, and suppressing the expression of virulence genes. Some phenolic derivatives, such as flavonoids and tannins, interfere with pathogen metabolism and protein biosynthesis, while others, like lignins, contribute to the physical reinforcement of cell walls, creating a barrier against microbial penetration [9]. Additionally, phenolic compounds can generate free radicals that induce oxidative damage in pathogens, compromising their cell wall integrity and DNA stability. Their role in plant immunity extends to the activation of defense-related genes, including those encoding pathogenesis-related (PR) proteins, and the initiation of hypersensitive response-mediated cell death, effectively limiting pathogen spread [10]. Furthermore, phenolic compounds serve as potent chemical deterrents against herbivory due to their bitter taste, toxicity, and ability to interfere with insect digestion and metabolism. Some compounds form complexes with dietary proteins, reducing their availability to herbivores, while others exhibit direct insecticidal activity. These properties contribute to plant resilience by minimizing feeding damage and enhancing survival under herbivore pressure [11,12]. Their biosynthesis, accumulation, and spatial distribution are tightly regulated, varying according to the plant’s developmental stage, tissue type, and environmental conditions [6,7]. A major group of phenolic compounds is the phenylpropanoids, which include flavonoids, anthocyanins, lignins, phytoalexins, and salicylic acid. These metabolites are synthesized in plants through interconnected metabolic pathways, primarily the pentose phosphate pathway, the shikimate pathway, and the phenylpropanoid pathway [13,14]. Phenolic compounds exhibit a wide range of structural diversity, which determines their specific biological functions. While some phenolic compounds are ubiquitous across many plant species, others are restricted to specific taxa, reflecting evolutionary adaptations to distinct ecological pressures [15]. The biosynthesis of phenylpropanoids is initiated by phenylalanine ammonia-lyase (PAL), a key enzyme that catalyzes the non-oxidative deamination of phenylalanine to trans-cinnamate, which serves as the precursor for cinnamic acid and links primary metabolism to the production of secondary metabolites [16,17,18]. PAL is an inducible enzyme that responds to biotic and abiotic stresses [19]. Its activity increases significantly in response to diverse stimuli, including tissue wounding, pathogen attacks, exposure to light, low temperatures, and hormonal signals [20,21].

Sweet basil (*Ocimum basilicum* L.), a widely known medicinal and aromatic plant from the Lamiaceae family, is rich in essential oils and phenolic compounds and is extensively used in traditional medicine [22]. *O. basilicum* is particularly rich in a wide variety of phenolic compounds. These include well-known phenolic acids such as rosmarinic acid, chicoric acid, vanillic acid, p-coumaric acid, benzoic acid, hydroxybenzoic acid, syringic acid, ferulic acid, protocatechuic acid, caffeic acid, and gentisic acid [23,24,25].

Plant growth-promoting rhizobacteria (PGPR) constitute a group of beneficial soil-borne microorganisms widely studied because of their ability to improve plant growth and development by producing phytohormones and providing better nutrient uptake [26]. Among the PGPR group, species from the genera *Bacillus* and *Pseudomonas* have been the most extensively studied for their effects on herbivorous insects, with all such effects being mediated through the plant. These bacteria enhance the plant’s defensive responses, demonstrating significant potential for application in pest management strategies [27].

*Bacillus* species are notable for their ability to form resilient spores, allowing them to persist in the soil for extended periods, even under adverse environmental conditions. These bacteria promote plant growth through various mechanisms, including the induction of systemic resistance (ISR), antibiosis, and competitive exclusion. As a result, microbial applications of *Bacillus* can enhance plant defense against biotic stressors and improve tolerance to environmental stress [28]. Moreover, the activation of ISR by *Bacillus* species is often regulated by the jasmonic acid (JA)/ethylene signaling pathway and, to a lesser extent, by salicylic acid (SA) [29]. This regulation activates downstream plant defenses and plays a role in mediating tritrophic interactions in some plants. *Bacillus* spp. also elicit the expression of JA-related genes and simultaneously upregulate genes involved in the production of SMs, such as allelochemicals that inhibit pest larval growth [30], further enhancing the plant’s ability to defend itself against herbivorous insects. For instance, when applied to cotton plants, PGPR induce systemic resistance and increase the production of gossypol, an SM that reduces the feeding and development of *Spodoptera exigua* [30]. Furthermore, the inoculation with PGPR can stimulate the production of plant volatiles, which attract predatory earwigs (Dermaptera) in response to herbivore attacks [31]. However, the effectiveness of PGPR in protecting the host plant greatly depends on the specific combination of PGPR and the herbivore species involved [32].

*Bacillus amyloliquefaciens* GB03, initially identified as a strain of *B. subtilis* [33], employs a variety of strategies to combat pathogen-induced diseases and enhance plant health. It produces a range of bioactive compounds, including SMs, phytohormones, cell wall-degrading enzymes, and antioxidants, which contribute to both pathogen suppression and plant defense enhancement. Additionally, *B. amyloliquefaciens* improves nutrient availability by solubilizing phosphorus and producing siderophores, which not only support plant growth but also inhibit pathogen development. Furthermore, it has been documented that inoculation with GB03 increases fresh weight in aromatic plants such as *Mentha piperita* [34] and *O. basilicum* [35]. Moreover, GB03 releases volatile compounds that enhance both fresh weight and essential oil accumulation in *O. basilicum* [35]. This species further enhances plant tolerance to environmental stress by triggering the expression of stress-related genes and stimulating the production of phytohormones and metabolites [27]. Research on plant–microorganism and plant–insect interactions has traditionally been conducted independently, with limited focus on their combined effects. PGPR have been shown to activate defense mechanisms in plants in a manner similar to how phytopathogens or herbivorous insects do [36]. Systemic defense involves the activation of the host plant’s enzymatic and protein machinery, leading to a reduction in the severity of damage caused by biotic stress [37,38].

In this study, we investigate the impact of herbivory by *Spodoptera frugiperda* larvae, inoculation with the PGPR *B. amyloliquefaciens* GB03, and their combination on the phenolic compounds content (TPC) in *O. basilicum* plants. Additionally, we analyze the levels of endogenous phytohormones and PAL activity, a key enzyme involved in plant defense responses. This integrative approach provides a more comprehensive understanding of how these biotic factors interact and influence plant biochemical pathways.

## 2. Results

### 2.1. Total Phenolic Content (TPC)

**Figure 1:**
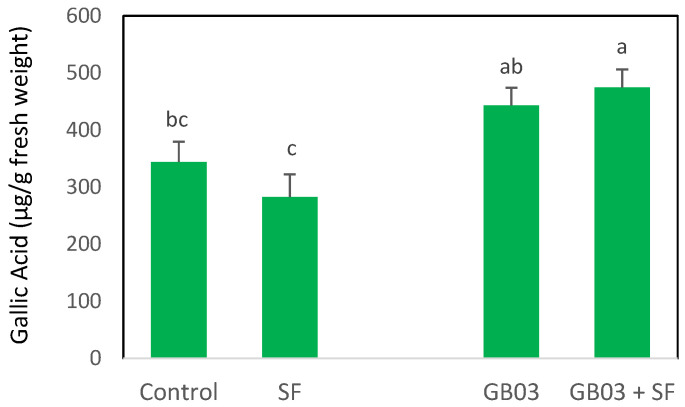
Total phenolic content, expressed as gallic acid equivalents, in *Ocimum basilicum* plants exposed to herbivory by *Spodoptera frugiperda* larvae (SF) and/or inoculated with *Bacillus amyloliquefaciens* GB03 (mean ± SE, *n* = 30). Letters above bars indicate significant differences according to Fisher’s LSD test (*p* < 0.05). Biological replicates *n* = 30 (Control), *n* = 29 (SF), *n* = 25 (GB03), *n* = 24 (GB03 + SF).

The accumulation of phenolic compounds in the *O. basilicum* plants inoculated with GB03 showed a slight increase compared to the non-inoculated plants; however, this increase was not statistically significant (*p* > 0.05) (Figure 1). The plants damaged by *S. frugiperda* larvae (SF) showed no significant differences in TPC compared to controls (*p* > 0.05), despite a slight reduction. The inoculated plants infested with *S. frugiperda* showed similar TPC values to the plants that were only inoculated (*p* > 0.05). However, their TPC was 60% and 40% higher than in the non-inoculated plants exposed to *S. frugiperda* and control plants, respectively (*p* < 0.05). There was no significant interaction between herbivory and inoculation (*p* > 0.05).

### 2.2. Phenylalanine Ammonia-Lyase Activity (PAL)

**Figure 2:**
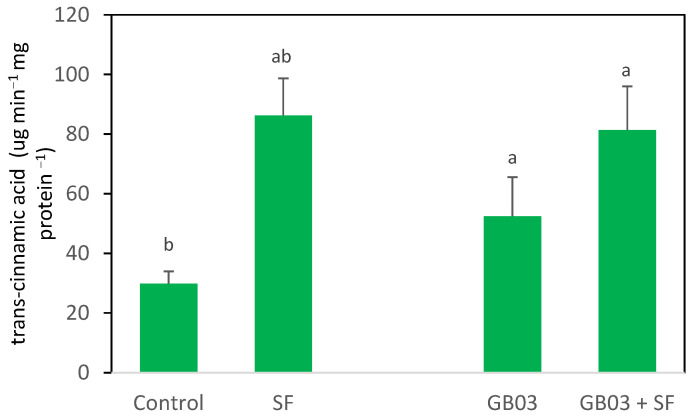
PAL activity in *Ocimum basilicum* inoculated with *Bacillus amyloliquefaciens* GB03, and/or damaged by *Spodoptera frugiperda* larvae (SF) (mean ± SE, *n* = 25). PAL activity is expressed as specific activity (µg min^−1^ mg protein^−1^), which represents the amount of trans-cinnamic acid produced per minute per milligram of protein. Letters above bars indicate significant differences according to Fisher’s LSD test (*p* < 0.05). Biological replicates *n* = 25 (Control), *n* = 24 (SF), *n* = 25 (GB03), *n* = 24 (GB03 + SF).

PAL activity was not significantly affected by the inoculation with GB03 compared to the control plants (*p* > 0.05). However, PAL activity increased 2.6-fold in the *O. basilicum* plants damaged by SF larvae compared to the control plants (*p* < 0.05) (Figure 2). When PGPR inoculation and SF infestation were combined, the increase in PAL activity was similar to that observed in the plants infested solely with the larvae (*p* > 0.05).

### 2.3. Endogenous Phytohormones

**Figure 3:**
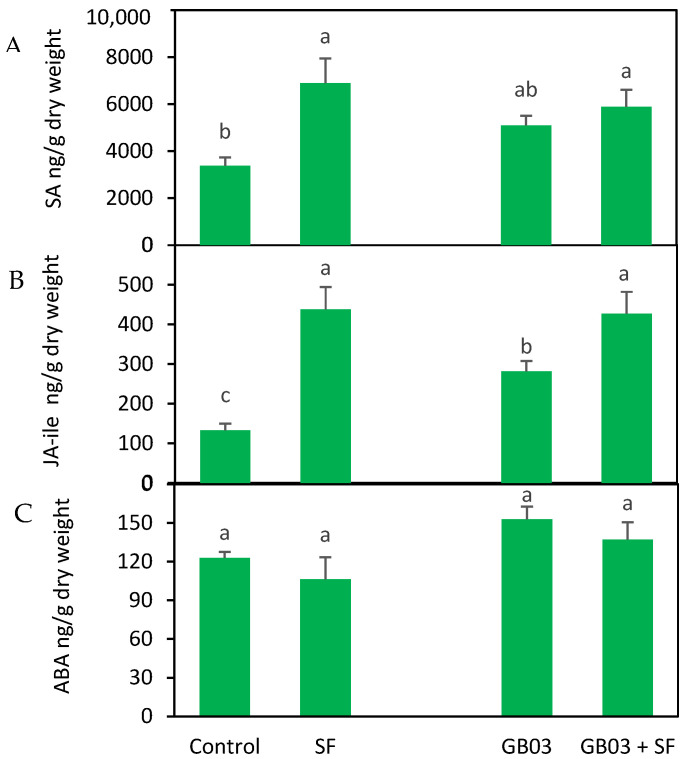
Endogenous phytohormone content, (**A**) salicylic acid (SA), (**B**) jasmonic acid–isoleucine (JA-ile), (**C**) abscisic acid (ABA), in *Ocimum basilicum* plants inoculated with *Bacillus amyloliquefaciens* GB03 and/or infested with *Spodoptera frugiperda* (mean ± SE, *n* = 8). Different letters indicate significant differences between treatments based on Fisher’s LSD test (*p* < 0.05).

When SA levels were measured 48 h after larval damage, no statistically significant differences were observed between the inoculated and non-inoculated plants, showing that SA levels remained similar in the insect-damaged plants regardless of inoculation (*p* > 0.05). However, a notable increase was detected in the plants damaged by SF larvae, regardless of inoculation status (*p* < 0.05), with concentrations approximately doubling compared to the control plants, particularly in those solely damaged by insects (Figure 3A). Endogenous levels of JA-ile were similar across the different treatments evaluated. Notably, the inoculated plants exhibited increases of approximately 35% for JA-ile compared to the non-inoculated plants (*p* < 0.05) (Figure 3B). Both the inoculated and non-inoculated plants damaged by SF larvae exhibited elevated JA-ile concentrations compared to the control plants. Specifically, JA-ile levels rose by approximately 70% in the plants that were either damaged by larvae alone or both inoculated and damaged (*p* < 0.05). Additionally, the plants that were only inoculated showed a 30% increase in JA levels (*p* < 0.05) compared to the control. Notably, JA-ile concentrations were similar between the plants solely damaged by the larvae and those both inoculated and damaged (*p* > 0.05). No significant differences were observed in abscisic acid (ABA) content across the different treatments evaluated (*p* > 0.05) (Figure 3C).

### 2.4. Principal Component Analysis

**Figure 4:**
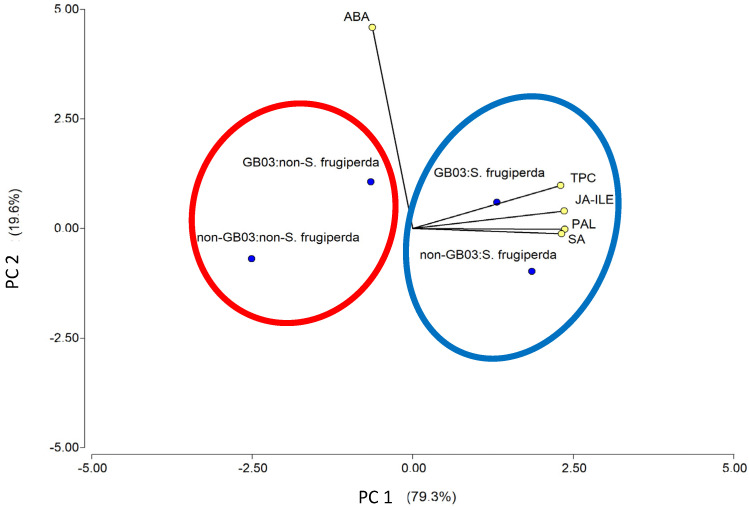
Principal component analysis for the physiological response of *O. basilicum* to *S. frugiperda* larvae herbivory and PGPR inoculation (GB03). TPC: total phenolic content, JA-ile: jasmonic acid, SA, salicylic acid, and PAL: phenylalanine ammonia-lyase activity.

In order to relate the treatments (herbivory and inoculation conditions) with the factors evaluated (TPC, PAL activity, and endogenous phytohormone levels, ABA, JA-ile, SA), a multivariate principal component analysis (PCA) was performed. This type of analysis provides a graph that facilitates the visualization and interpretation of the data set and the variables. In the PCA (Figure 4), for the insect herbivory and inoculation conditions, PC1 accounted for 79.3%, and PC2 for 19.6%, of the total variability in the data. Together, both axes explained 98.9% of the total variance and provided a cophenetic correlation coefficient of 0.999, indicating an excellent preservation of the original distance relationships in the reduced-dimensional space. We observed a strong positive correlation (acute angle) between TPC and PAL activity, JA-ile and SA, while the location on the plot of ABA showed a less pronounced angle, indicating a weaker correlation. This plot demonstrates that plants damaged by the SF larvae, regardless of whether they are inoculated or not, are located in proximity to almost all the evaluated variables (blue circle). Specifically, closer proximity to TPC is observed when larval damage is combined with inoculation. On the other hand, the plants not damaged by SF larvae, regardless of inoculation, were located on the plot far from the evaluated variables (red circle), indicating a lower overall effect. Specifically, the non-inoculated plants without herbivory were the most distant, suggesting that the effects of the treatments were minimal, considering the absence of both herbivory and inoculation. In contrast, the inoculated plants without herbivory were positioned somewhat closer to the evaluated variables, indicating a slight effect on the parameters studied, showing a modest response to inoculation alone.

## 3. Discussion

### 3.1. Rhizobacteria-Mediated Plant Defense and Stress Response

The rhizosphere microbiome plays a crucial role in promoting crop health and productivity. Previous studies, including those conducted by our research group, have demonstrated that PGPR colonization can significantly enhance growth in medicinal and aromatic plants [34,39,40,41,42]. Additionally, PGPR is known to induce ISR, particularly in response to herbivory [26,43,44,45,46,47]. This mechanism is activated through the stimulation of JA and ET signaling pathways, which enhance the plant’s defenses against both pathogens and insect pests [46,48].

### 3.2. Phenolic Compound Accumulation and Biosynthesis Pathways

Phenolic compounds are involved in plant defense against microbial pathogens and insect herbivores [9,10,49]. While PGPR-induced increases in TPC have been reported in several aromatic plant species [50,51], our findings showed no significant difference in TPC between *O. basilicum* plants inoculated with GB03 and non-inoculated. This contrasts with previous studies, such as those on *Tagetes minuta*, where inoculation with *Pseudomonas fluorescens* and *Azospirillum brasilense* resulted in a twofold increase in TPC [42], and on *Mentha piperita*, where GB03 inoculation significantly enhanced phenolic accumulation [50]. These discrepancies suggest that the impact of rhizobacterial inoculation on TPC is species-dependent and influenced by the plant–microbe interaction.

No significant difference in TPC was observed in *O. basilicum* plants damaged by *S. frugiperda* compared to controls; however, this contrasts with findings in *M. piperita*, where *Rachiplusia nu* herbivory significantly increased TPC levels [52]. Similarly, *Arachis hypogaea* genotypes exhibited higher TPC following feeding damage by *Helicoverpa armigera* [29]. Despite the lack of significant changes in TPC, *O. basilicum* contains key phenolic compounds such as caffeic acid, sinapic acid, rosmarinic acid, and ferulic acid, which reduce digestibility, lower nutritional quality, and act as chemical deterrents against herbivores [23,53]. Additionally, rosmarinic acid has demonstrated insecticidal properties, significantly increasing mortality rates in *Culex quinquefasciatus* mosquito larvae and adults [54]. The specific defensive responses triggered depend on the herbivore species, pathogenic interactions, and the type of phenolic compounds involved [49].

While PGPR inoculation did not increase TPC in *O. basilicum*, it did lead to a significant increase in EO yield, as observed in previous studies [55]. Additionally, *S. frugiperda* larvae feeding on GB03-inoculated plants were negatively affected, showing reduced pupal size and lower adult emergence [56]. These findings highlight the complexity and plasticity of plant defense responses, where both phenolic compounds and EO contribute in complementary ways to biotic stress resistance.

Regarding PAL activity, in our study, PGPR inoculation alone did not lead to a significant increase in PAL activity in *O. basilicum* plants. However, previous research has shown that PGPR inoculation can enhance PAL activity in other plant species. For instance, Cappellari et al. [50] documented a rise in PAL activity in *M. piperita* plants inoculated with different PGPR strains, correlating with an increase in TPC. Similarly, Joni et al. [57] observed enhanced PAL activity in tomato plants inoculated with *Pseudomonas corrugata* or *P. aureofaciens* compared to control plants. These discrepancies suggest that the induction of PAL activity may vary depending on the plant species, PGPR strain, or experimental conditions.

The increase in PAL activity observed in herbivore-damaged plants in our study aligns with previous research showing similar responses in plants subjected to insect feeding. Rani and Pratyusha [58] reported a rise in PAL levels in cotton plants fed on by *S. litura*, while Kovalikova et al. [59] observed elevated PAL activity and TPC in *Brassica oleracea* plants damaged by *Pieris brassicae*, a lepidopteran herbivore specialized in Brassicaceae. Similarly, Sandhyarani and Rani [60] found concurrent increases in both total phenolics and PAL activity in *Ipomoea batatas* following *S. litura* herbivory. However, contrasting results have been reported for *Arachis hypogaea*, where herbivory by three chewing lepidopterans—*Amsacta albistriga, Aproaerema modicella*, and *Spilosoma obliqua*—led to an increase in total phenolics but did not significantly affect PAL activity [61].

This increase observed in PAL activity was generally associated with higher TPC, except in plants that were only insect-damaged, where PAL activity rose without a corresponding increase in phenolic content. This discrepancy may be due to the presence of other phenolic-derived compounds in sweet basil, particularly eugenol, a phenylpropanoid found in EOs. Previous studies from our group have reported an increase in eugenol content in insect-damaged plants [55]. It is important to note that the Folin–Ciocalteu method used to determine TPC quantifies only water-soluble phenols, excluding lipophilic compounds like eugenol. Therefore, the observed increase in PAL activity in insect-affected plants may reflect an elevation in eugenol content rather than an increase in water-soluble phenolics.

### 3.3. Role of Phytohormones in Plant Defense Modulation

Phytohormones such as JA and SA play pivotal roles in modulating plant responses to biotic stressors, including rhizobacterial colonization and insect herbivory [62,63]. In our study, JA levels increased in all treatments compared to control plants, consistent with previous reports in cotton plants, where PGPR inoculation resulted in a fourfold rise in JA content [30]. Similarly, inoculated *Arabidopsis thaliana* plants exhibited enhanced JA-related gene expression [64]. In contrast, *Vicia faba* plants inoculated with *B. amyloliquefaciens* did not show significant changes in JA levels [65].

Herbivory by *S. frugiperda,* in the present study, led to a significant increase in both JA and SA levels, consistent with findings in *Arabidopsis* and *Brassica* species subjected to lepidopteran feeding [66,67]. This supports the role of these phytohormones in mediating ISR, which enhances plant defense against insect herbivory.

The combined effects of PGPR inoculation and herbivory play a crucial role in shaping plant defense strategies, as observed in our study, where JA and SA levels increased in response to both factors compared to control plants. Kousar et al. [68] reported that plants inoculated with *B. endophyticus* and *P. aeruginosa* exhibited increased SA and JA levels when exposed to chewing larvae compared to control plants. Similarly, Zebelo et al. [30] observed elevated JA levels in cotton plants inoculated with PGPR and subjected to *S. exigua* herbivory. Supporting our results, *Mentha piperita* plants inoculated with strain GB03 and exposed to *Rachiplusia nu* showed higher levels of both JA and SA [52]. Additionally, enhanced expression of JA and ethylene signaling pathways, including the ORA59 pathway, has been demonstrated to play a crucial role in mediating resistance against herbivory [66]. The ability of PGPR to activate JA-dependent signaling pathways aligns with our findings, reinforcing the concept of PGPR-induced priming as a key mechanism in plant defense [29,46].

These findings reinforce the role of PGPR in modulating phytohormone-mediated defenses, particularly through JA and SA signaling. The enhanced accumulation of these phytohormones in response to the combined stressors suggests that PGPR inoculation strengthens plant adaptive mechanisms, enabling a more robust and efficient defense activation against herbivory.

### 3.4. Interactions Between PGPR Inoculation and Herbivory in Defense Mechanisms

ISR plays a fundamental role in plant defense, particularly when mediated by PGPR in conjunction with herbivory. PGPR are known to activate ISR by stimulating JA signaling pathways, thereby enhancing the plant’s ability to defend against pathogens and insect pests [69]. This process strengthens plant defense responses by increasing the production of SMs, such as phenolic compounds, which are key in plant protection [69].

Both SA and JA play critical roles in modulating PAL activity, which leads to the accumulation of phenolic compounds. JA has been shown to increase PAL activity and induce the expression of phenylpropanoid-related genes, promoting the accumulation of phenolic compounds, as observed in radish sprouts [70] and *Agastache rugosa* [71]. In *Larix gmelinii*, PAL gene expression was significantly higher in JA-treated plants compared to control plants [72]. Furthermore, JA elicitation led to a rapid increase in phenolic compound concentration in cell cultures of *Hypericum perforatum* [73] and induced a greater accumulation of phenolic compounds in *Brassica rapa* compared to ABA and SA treatments [74].

Similarly, SA also regulates PAL activity by modulating the expression of the PAL gene [75], promoting the accumulation of phenolic compounds such as chlorogenic acid, caffeic acid, p-coumaric acid, ferulic acid, and gallic acid [76,77]. In tomatoes, SA plays an important role in phenolic compound accumulation [78], and, in tobacco, it induces the expression of genes involved in the synthesis of scopoletin [79].

These findings collectively highlight the crucial roles of JA and SA in enhancing PAL activity and phenolic compound biosynthesis, which are essential components of plant defense mechanisms. The synergistic effects of PGPR inoculation and herbivory on these pathways further emphasize the complexity of the regulatory networks governing plant responses to biotic stress. Furthermore, the ability of PGPR to *prime* plant defenses suggests that microbial inoculation may prepare plants for faster and stronger activation of defense responses upon herbivore attack.

## 4. Materials and Methods

### 4.1. Bacterial Strains, Culture Conditions and Media

The PGPR strain utilized in this study was *Bacillus amyloliquefaciens* GB03. It was cultured on Luria–Bertani (LB) medium and preserved in nutrient broth with 15% glycerol at −80 °C for long-term storage. For experimental purposes, the bacteria were cultured on nutrient agar. Single colonies were then transferred to 100 mL flasks containing the LB (Luria–Bertani) medium and grown aerobically on a rotating shaker (150 rpm) for 24 h at 28 °C. The resulting bacterial suspension was diluted in sterile saline solution (0.9% sodium chloride, NaCl) to achieve a final concentration of 10^9^ colony-forming units (CFU) per milliliter. Subsequently, 1 mL of this suspension was applied around the base stem of the plants. The control plants were inoculated with 1 mL of saline solution.

### 4.2. Insects Culture

The *Spodoptera frugiperda* second-instar larvae used in this study were provided by the AgIdea (Agricultural Innovation Applied Research-Argentina) company and were obtained from a colony without previous insecticide exposure. The colony has been maintained under these conditions for six months, with larvae reared on a semi-synthetic diet [80] in a 23–25 °C chamber with 70% humidity, under a 16:8 h light/dark photoperiod.

### 4.3. Seed Sterilization and Plant Cultivation

Seeds of *Ocimum basilicum* L. var. Genovese (Florensa Argentina S.A., Córdoba, Argentina) were surface sterilized by soaking for 2 min in 70% (*v*/*v*) ethanol and for 20 min in 1% (*v*/*v*) sodium hypochlorite. After this, they were thoroughly rinsed four times with sterile distilled water and placed in plastic pots filled with sterilized vermiculite that had been sterilized by autoclaving. Following a 15-day period, the plantlets were transplanted into larger plastic pots (12 cm × 22 cm) filled with sterilized vermiculite. They were grown in a growth chamber under controlled conditions of light (16/8 h light/dark cycle), temperature (22 ± 2 °C), and relative humidity (~70%) and watered every week with 20 mL of Hoagland solution per pot. After seven days, the plants were inoculated with 1000 μL of bacterial suspension or with saline solution in the case of control plants. The experiments were conducted three times (10 pots per treatment, one plant per pot), and arranged randomly in the growth chamber.

### 4.4. Bioassays and Treatments

After 45 days post-inoculation, each plant was subjected to a 4-h exposure to three previously starved *Spodoptera frugiperda* larvae. This test was conducted within entomological cages. Following 48 h of damage, vegetal material was harvested, weighed, and transferred to liquid nitrogen for subsequent analysis. The experimental treatments included the following: (a) control (non-inoculated plants), (b) plants inoculated with *B. amyloliquefaciens* GB03 (GB03), (c) plants infested with *S. frugiperda* (SF), and plants inoculated with *B. amyloliquefaciens* GB03 and infested with *S. frugiperda* (SF + GB03).

### 4.5. Determination of Total Phenolic Content (TPC)

150 mg of fresh plant tissue (leaves) were weighed and homogenized with 5 mL of distilled water. After resting for 24 h in the dark, the plant extract was prepared. Total phenolic values were determined using the Folin–Ciocalteu reagent [81]. Each plant extract (0.5 mL) or gallic acid (1 mg/mL) (standard phenolic compound) was mixed with Folin–Ciocalteu reagent (0.5 mL, diluted with 8 mL distilled water) and aqueous Na_2_CO_3_ (1 mL, 1 M). After 1 h, the content of TPC was determined by colorimetry at a wavelength of 760 nm. Total phenolic values were expressed in terms of µg gallic acid (a common reference compound) equivalent per g fresh plant weight [50].

### 4.6. Determination of PAL Enzyme Activity

PAL was extracted from 100 mg leaves; plant material was homogenized with liquid nitrogen using a mortar and pestle containing appropriate buffer solution (50 mM potassium phosphate and 1 mM EDTA, pH 7.8) and 1% PVP (polyvinylpyrrolidone) and then filtered through a 0.20 mm nylon filter into a centrifuge tube. The tissue extract was centrifuged at 10,000 rpm for 20 min at 4 °C. The supernatant to be used for enzymatic activity determination was stored at 20 °C. Protein concentration was determined using the method described by Bradford [82].

### 4.7. Hormone Extraction

Phytohormones were extracted from 50 mg homogenized material using three rounds of extraction in 80% methanol, which was acidified to pH 2.4 with hydrochloric acid. For round 1, 400 µL, for round 2, 200 µL, and, for round 3, 100 µL solvent was used. Cell extraction was performed in 1.5 mL cryo-tubes with reinforced walls (Biozyme). In order to enhance cell rupture and extraction, one steel bead of 3 mm, three steel beads of 1 mm diameter, and glass beads of 0.75 to 1 mm diameter (Carl Roth GmbH) were added to each tube, and bead milling was performed for 3 × 1 min in a homogenizer (FastPrep24, MP Biomedicals, Santa Ana, CA, USA). The extraction solvent contained deuterated hormone analogs of known concentration (d6-ABA: 12.5 µg/L; 2(d2)-JA-ILE: 12.5 µg/L; d4-SA: 50 µg/L). The combined extracts were centrifuged and stored on ice until measurement on the same day [83].

### 4.8. SPE-UPLC-MS/MS Phytohormone Analysis

Phytohormones were separated using a Nucleoshell RP Biphenyl column (100 mm × 2 mm, 2.1 µm, Macherey und Nagel, Düren, Germany) with the following gradient: 0–2 min: 5% B; 13 min: 95% B; 13–15 min: 95% B; 15–18 min: 5% B. The column temperature was set to 40 °C. Solvent A consisted of 0.3 mM ammonium formate, acidified with formic acid to pH 3.0, while solvent B was acetonitrile. The autosampler temperature was maintained at 4 °C. For sample preparation, a prototype solid-phase extraction (SPE) system was used in conjunction with UPLC. Per sample, 600 µL of plant extract was injected onto a divinylbenzene micro-SPE stationary phase (30 mg, SparkHolland B.V., Emmen, The Netherlands) at a flow rate of 200 µL min^−1^. Phytohormones were retained by the simultaneous addition of excess water (3800 µL min^−1^). Transfer from the SPE cartridge to the UPLC column was performed using 120 µL of a solution containing 20% acetonitrile and 80% solvent A. The entire procedure was carried out on a prototype system composed of a CTC Combi-PAL autosampler (1 mL injection loop), an ACE 96-well plate SPE unit, a high-pressure dispenser, an SPH1299 UPLC gradient pump, an EPH30 UPLC dilution pump, and a Mitral column oven (all from Axel-Sembrau GmbH, Sprockhövel, Germany). Phytohormones were detected by mass spectrometry using a QTrap 6500 (Sciex, Framingham, MA, USA) with electrospray ionization in positive mode. Detection was performed by multiple reaction monitoring (MRM) with a 5 ms dwell time per transition. The ion source was heated to 450 °C. The curtain gas was set to 35 psi, while ion source gases GS1 and GS2 were set to 60 psi and 70 psi, respectively. The electrospray ion spray voltage was 5500 V.

### 4.9. Statistical Analysis

The data were pooled and subjected to analysis of variance (ANOVA) followed by a comparison of multiple treatment levels with controls using Fisher’s post-hoc LSD (least significant difference) test. Differences between means were considered significant for *p* values < 0.05. The Infostat software program, v. 2020 (Group Infostat, Universidad Nacional de Córdoba, Argentina) was used for all statistical analyses. The ANOVA tables with detailed results are provided in the Supplementary Materials.

## 5. Conclusions

The findings of this study highlight that sweet basil plants exhibit selective and differentiated defense responses to various stress stimuli. PGPR inoculation and herbivory by *S. frugiperda* larvae independently elicited distinct defensive mechanisms, particularly in relation to phenolic compound accumulation. While herbivory alone did not lead to a significant increase in TPC, the combination of PGPR inoculation and herbivory resulted in a notable enhancement of phenolic accumulation. This suggests that phenolic compounds play a supplementary role in response to insect damage, with a more prominent involvement in plants subjected to both biotic stresses. The activation of JA and SA signaling pathways was evident across all treatments, but the increase in phenolic compounds required an interaction between rhizobacterial inoculation and herbivory. Additionally, PGPR inoculation contributed to a consistent baseline of defense, suggesting a robust induction of ISR that primes plants for heightened defensive responses even under combined biotic stresses. These findings suggest that integrating PGPR inoculation into pest management strategies could enhance crop resilience, potentially reducing reliance on chemical interventions. To further validate these findings, field studies are recommended to assess how these interactions manifest in more natural, complex environments. Moreover, exploring the long-term effects of combined treatments on plant growth, yield, and overall health would provide a deeper understanding of their practical applications in sustainable agriculture.

## Data Availability

Data are contained within the article and Supplementary Materials.

## Supplementary Materials

The following supporting information can be downloaded at: https://www.mdpi.com/article/10.3390/plants14060857/s1. Table S1. Results for the ANOVA analysis of total phenolic content, expressed as gallic acid equivalents, in Ocimum basilicum plants exposed to herbivory by *Spodoptera frugiperda* larvae (SF) and/or inoculated with Bacillus amyloliquefaciens GB03; Table S2. Results of ANOVA for phenylalanine ammonia-lyase (PAL) activity in Ocimum basilicum plants inoculated with Bacillus amyloliquefaciens GB03 and/or damaged by *Spodoptera frugiperda* larvae (SF). Table S3. Results of ANOVA for endogenous phytohormone content (A) salicylic acid (SA), (B) jasmonic acid-isoleucine (JA-Ile), and (C) abscisic acid (ABA) in Ocimum basilicum plants inoculated with Bacillus amyloliquefaciens GB03 and/or infested with *Spodoptera frugiperda*.
