# Supplementary material for: Influence of Plant Growth-Promoting Rhizobacteria (PGPR) Inoculation on Phenolic Content and Key Biosynthesis-Related Processes in Ocimum basilicum Under Spodoptera frugiperda Herbivory
Source: Plants (Basel). 2025 Mar 10;14(6):857. doi: 10.3390/plants14060857 (PMC11944467; doi:10.3390/plants14060857)
Supplement: Supplementary file 1 [file plants-14-00857-s001.zip › plants-3500714-supplementary.pdf]

Supplementary Table S1. Results for the ANOVA analysis of total phenolic content, expressed as gallic acid equivalents, in *Ocimum basilicum* plants exposed to herbivory by *Spodoptera frugiperda* larvae (SF) and/or inoculated with *Bacillus amyloliquefaciens* GB03

Source	SS	df	MS	F	p
Model	514648,08	3	171549,36	6,85	0,0003
PGPR	439017,72	1	439017,72	17,52	0,0001
INSECT	45766,89	1	45766,89	1,83	0,1795
PGPR*INSECT	35988,68	1	35988,68	1,44	0,2335
Error	2631317,17	105	25060,16		
Total	3145965,25	108			

Supplementary Table S2. Results of ANOVA for phenylalanine ammonia-lyase (PAL) activity in *Ocimum basilicum* plants inoculated with *Bacillus amyloliquefaciens* GB03 and/or damaged by *Spodoptera frugiperda* larvae (SF).

Source	SS	df	MS	F	p
Modelo	23789,08	3	7929,69	3,05	0,0392
PGPR	871,11	1	871,11	0,34	0,5659
INSECT	20418,91	1	20418,91	7,85	0,0077
PGPR*INSECT	2127,36	1	2127,36	0,82	0,3710
Error	106602,66	94	2600,06		
Total	130391,75	98			

Supplementary Table S3. Results of ANOVA for endogenous phytohormone content (A) salicylic acid (SA), (B) jasmonic acid-isoleucine (JA-Ile), and (C) abscisic acid (ABA) in *Ocimum basilicum* plants inoculated with *Bacillus amyloliquefaciens* GB03 and/or infested with *Spodoptera frugiperda.*

A)

Source	SS	df	MS	F	p
Modelo	46500642,86	3	15500214,29	4,67	0,0104
PGPR	871557,14	1	871557,14	0,26	0,6130
INSECT	32572857,14	1	32572857,14	9,82	0,0045
PGPR*INSECT	13056228,57	1	13056228,57	3,94	0,0588
Error	79622000,00	24	3317583,33		
Total	126122642,86	27			

B)

Source	SS	DF	MS	F	p
Modelo	434342,86	3	144780,95	11,65	0,0001
PGPR	32914,29	1	32914,29	2,65	0,1167
INSECT	356628,57	1	356628,57	28,70	<0,0001
PGPR*INSECT	44800,00	1	44800,00	3,60	0,0697
Error	298257,14	24	12427,38		
Total	732600,00	27			

C)

Source	SS	df	MS	F	p
Modelo	8852,89	3	2950,96	2,58	0,0762

PGPR	6698,21	1	6698,21	5,85	0,0232
INSECT	1887,15	1	1887,15	1,65	0,2109
PGPR*INSECT	1,44	1	1,44	1,3E-03	0,9720
Error	28616,07	25	1144,64		
Total	37468,97	28			
